# Cell Cycle Genes Are the Evolutionarily Conserved Targets of the E2F4 Transcription Factor

**DOI:** 10.1371/journal.pone.0001061

**Published:** 2007-10-24

**Authors:** Caitlin M. Conboy, Christiana Spyrou, Natalie P. Thorne, Elizabeth J. Wade, Nuno L. Barbosa-Morais, Michael D. Wilson, Arindam Bhattacharjee, Richard A. Young, Simon Tavaré, Jacqueline A. Lees, Duncan T. Odom

**Affiliations:** 1 Cancer Research UK-Cambridge Research Institute, Li Ka Shing Centre, Cambridge, United Kingdom; 2 Statistical Laboratory, Department of Pure Mathematics and Mathematical Statistics, University of Cambridge, Cambridge, United Kingdom; 3 Department of Applied Mathematics and Theoretical Physics, University of Cambridge, Cambridge, United Kingdom; 4 Department of Ecology and Evolutionary Biology, University of Connecticut, Storrs, Connecticut, United States of America; 5 Agilent Technologies, Andover, Massachusetts, United States of America; 6 Department of Biology, Massachusetts Institute of Technology, Cambridge, Massachusetts, United States of America; 7 Whitehead Institute for Biomedical Research, Cambridge, Massachusetts, United States of America; Pasteur Institute, France

## Abstract

Maintaining quiescent cells in G0 phase is achieved in part through the multiprotein subunit complex known as DREAM, and in human cell lines the transcription factor E2F4 directs this complex to its cell cycle targets. We found that E2F4 binds a highly overlapping set of human genes among three diverse primary tissues and an asynchronous cell line, which suggests that tissue-specific binding partners and chromatin structure have minimal influence on E2F4 targeting. To investigate the conservation of these transcription factor binding events, we identified the mouse genes bound by E2f4 in seven primary mouse tissues and a cell line. E2f4 bound a set of mouse genes that was common among mouse tissues, but largely distinct from the genes bound in human. The evolutionarily conserved set of E2F4 bound genes is highly enriched for functionally relevant regulatory interactions important for maintaining cellular quiescence. In contrast, we found minimal mRNA expression perturbations in this core set of E2f4 bound genes in the liver, kidney, and testes of *E2f4* null mice. Thus, the regulatory mechanisms maintaining quiescence are robust even to complete loss of conserved transcription factor binding events.

## Introduction

Quiescence of cellular proliferation is crucial for mammalian tissue homeostasis, and aberrant activation of cell cycle programs can lead to cancer [1], [2], [3], [4], [5]. In mammalian cells, the highly conserved, multi-subunit complex known as DREAM is principally responsible for inhibiting cellular proliferation [6], [7], [8], and DREAM member homologs can be found in drosophila, worms, and mammals [2], [4], [6], [7], [8], [9], [10], [11], [12]. The DREAM complex is composed of multiple subunits with different functional roles; for instance, the site-specific transcription factor E2F4 and the pocket protein p130 serve to anchor the DREAM complex to direct functional targets. Consistent with a global role in maintaining quiescence, E2F4 has been shown to bind to and regulate a set of proliferation and cell cycle related targets in a number of ex vivo human cell lines, including glioblastoma [8], [13], fibroblast [14], and osteoblasts [15]. However, it is not known whether E2F4 controls similar genes in primary, quiescent human tissues, nor whether these regulatory connections are conserved evolutionarily.

Removal of key E2F components of the multisubunit complexes that control the cell cycle can cause aberrant activation of cellular proliferation in specific tissues during development and in adulthood reviewed in ([2], also [5]). For instance, homozygous loss of E2f4 causes a dramatic reduction in erythropoiesis in fetal mouse liver [16], [17]. Gene expression analysis revealed broad, substantial changes in transcription between developing erythrocytes lacking E2f4 and their wild-type counterparts [17]. The tissue-restricted nature of this phenotype indicates the presence of overlapping and partially redundant roles for other E2F transcription factors [1], [2], [4], [18], [19], [20]; for instance, it is known that E2F5 and E2F6 can compensate for loss of E2F4 [21], [22].

Despite wide fluctuations and evolutionary turnover of transcription factor binding events between mouse and human [23], it has been proposed that conserved genomic occupancy of a transcription factor binding can enrich for functionally relevant regulatory connections [24]. By comparing E2F4 DNA-binding events among multiple primary tissues in human and mouse, we uncovered a conserved set of regulatory interactions potentially relevant to maintaining cellular quiescence. We further inspected the tissue-specific gene expression programs in *E2f4* null mice to determine the transcriptional importance of E2f4 binding in primary mouse tissues.

## Results

### E2F4 binds a common set of cell cycle genes in multiple primary human tissues

**Figure 1:**
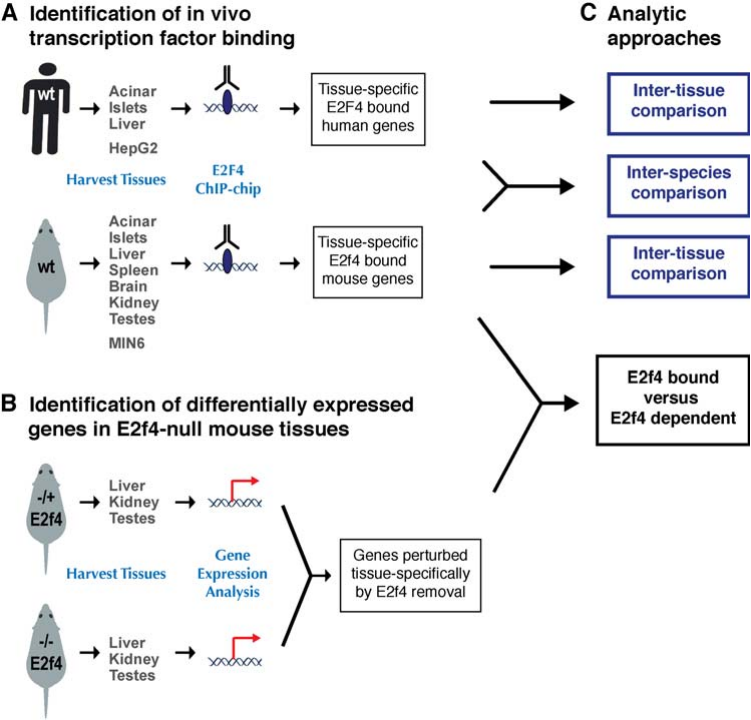
Strategy to compare E2F4 bound and E2F4 dependent gene expression in mouse and human. (A) Transcription factor binding was identified *in vivo* using chromatin immunoprecipitation combined with proximal promoter arrays in three human tissues, a human cell line, seven mouse tissues, and a mouse cell line. (B) Gene expression in the liver, kidney, and testes of littermate mice lacking one or two copies of E2f4 were compared in replicate, and genes specifically perturbed in the adult identified in all three tissues. (C) Analysis approaches identified the genes bound in common among all human and all mouse tissues, those shared between species, and whether any genes were both bound by E2f4 in mouse adult liver, kidney, or testes, and whose expression were altered by removal of E2f4.

We identified the proximal promoter regions that E2F4 occupies in three primary human tissues (hepatocytes, pancreatic acinar, and pancreatic islets) directly isolated from donor organs, and an asynchronous human cell line (HepG2), using chromatin immunoprecipitation and promoter microarrays representing 13,000 regions in the human genome [15], [25] (Figure 1A). Proximal promoter arrays targeted to transcription start sites capture the large majority of E2F4-chromatin interactions in the genome [8], [14]; we confirmed this result using whole-chromosome arrays that indicated that E2F4 binds largely at transcriptional start sites within the human and mouse genomes (Figure S1). For each tissue/species, assessment of genes bound by E2F4 was determined using a corrected p-value derived from empirical Bayes and linear model analysis across replicate microarrays for each gene [26] (Materials and Methods).

**Figure 2:**
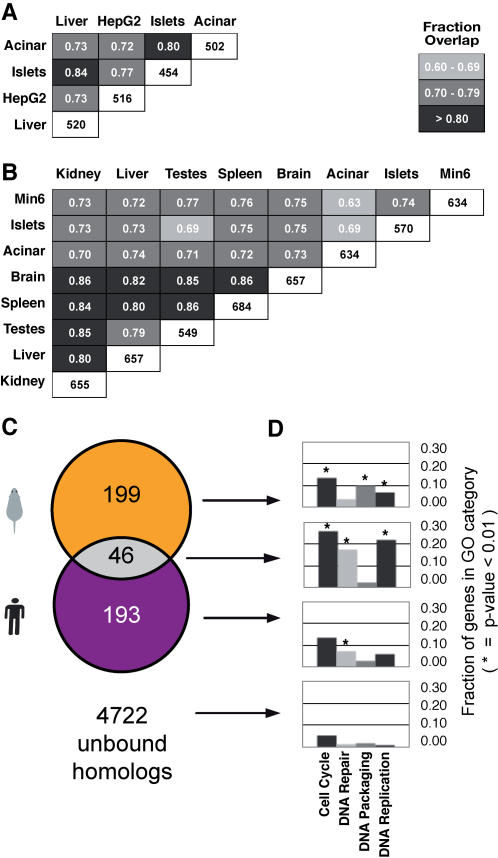
Genome-wide binding of E2F4 in mouse and human tissues. (A) Fraction of overlap of human genes bound by E2F4 in liver, pancreatic islets, pancreatic acinar, and HepG2 is shown as a grayscale shaded graph. Fractions and shading are calculated relative to the tissue with the fewest bound genes. The total number of binding targets for each tissue is shown in white boxes. (B) Similar plot for the overlap of mouse genes bound by E2f4 in liver, spleen, kidney, brain, testes, pancreatic islets, pancreatic acinar, and Min6. (C) Overlap between the mouse and human genes bound in common in all mouse and human tissues where homologs exist between the species. Consistent with previous results [23], the overlap is approximately 20%. (D) The genes bound by E2F4 in both mouse and human are substantially enriched in genes accounting for E2F4 function.

On our array platform, we found that E2F4 binds approximately 500 to 700 human genes, depending on the tissue. Among all three quiescent primary human tissues and the proliferating HepG2 carcinoma line, we observed overlap greater than 70% and as high as 84% (Figure 2A, Figure S2). This overlap is similar to the overlap previously observed between E2F4 bound genes in glioblastoma T98G cells and osteosarcoma U2OS cells on different microarray platforms under cell cycle arresting conditions [8]; our data capture approximately 75% of the same targets compared with the data from references [8], [15] (Materials and Methods).

A substantial majority of genes bound by E2F4 are bound in most tissues; we identified a core set of approximately 450 genes common to all human tissues in our study (Table 1, Figure S3, Materials and Methods). These include prior known E2F4 targets, including genes involved in cell cycle (CCNB1, INCENP, GSPT1, CDC6) and DNA repair (BRCA1, EXO1, XRCC1) [8], [13], [14], [15]. Analysis of gene ontology categories showed that cell cycle, proliferation, and DNA repair genes are consistently over-represented (Figure S3). Importantly, inspection of the tissue-specific targets revealed no functional pathway enrichment (Figure S4). We confirmed that the canonical E2F4 binding sequence was highly enriched in the E2F4 bound promoter regions. Typically we found 75% of the bound regions contained a known E2F4 binding sequence, compared with 35% among the unbound promoters present on the human promoter microarray (Figure S5). Taken together, our data indicate that, largely independent of the particular tissue-specific nuclear environment, E2F4 binds to and potentially regulates a similar set of human genes.

**Table 1:** Functional categorization of genes bound commonly among human tissues.

Selected genes (by category) bound by E2F4 in all human tissues			p-value for E2F4 binding			
Process	Gene	Full Name	Liver	Acinar	Islets	HepG2
**Cell Cycle**	ZWINT	ZW10 interactor	4.0E-08	6.0E-08	7.0E-08	4.0E-08
(1×10–44)	CCNB1	Cyclin B1	7.0E-08	9.0E-08	2.0E-07	8.0E-08
	INCENP	Inner centromere protein	2.0E-07	3.0E-07	3.0E-07	2.0E-07
	CDC23	Cell division cycle 23 homolog	2.0E-06	3.0E-06	3.0E-06	1.0E-06
	CDC6	Cell division cycle 6 homolog	5.0E-08	7.0E-08	9.0E-08	5.0E-08
	CDK2	Cyclin-dependent kinase 2	4.0E-06	8.0E-06	5.0E-06	5.0E-06
	NEK2	NIMA-related kinase 2	2.0E-05	3.0E-05	2.0E-05	2.0E-05
	MAD2L1	MAD2 mitotic arrest deficient-like 1	5.0E-07	1.0E-06	9.0E-07	5.0E-07
	CDC45L	CDC45 cell division cycle 45-like	3.0E-07	5.0E-07	5.0E-07	3.0E-07
	CCNB2	Cyclin B2	2.0E-08	2.0E-08	3.0E-08	3.0E-08
	RBL1	Retinoblastoma-like 1 (p107)	3.0E-06	4.0E-06	4.0E-06	3.0E-06
	NUDC	Nuclear distribution gene C homolog	3.0E-05	4.0E-05	3.0E-05	3.0E-05
	E2F3	E2F transcription factor 3	1.0E-05	1.0E-05	1.0E-05	1.0E-05
	TCF19	Transcription factor 19 (SC1)	9.0E-07	2.0E-06	2.0E-06	7.0E-07
	CCNG2	Cyclin G2	1.0E-07	1.0E-07	2.0E-07	1.0E-07
**DNA Replication**	RFC3	Replication factor C (activator 1) 3	1.0E-06	2.0E-06	2.0E-06	9.0E-07
(4×10–19)	PCNA	Proliferating cell nuclear antigen	2.0E-06	3.0E-06	3.0E-06	2.0E-06
	POLE3	Polymerase epsilon 3 (p17 subunit)	2.0E-05	2.0E-05	2.0E-05	2.0E-05
	NUP98	Nucleoporin 98 kDa	1.0E-05	2.0E-05	2.0E-05	2.0E-05
	ORC3L	Origin recognition complex, subunit 3-like	4.0E-07	5.0E-07	5.0E-07	3.0E-07
	RRM1	Ribonucleotide reductase M1 polypeptide	2.0E-07	3.0E-07	3.0E-07	2.0E-07
	ORC1L	Origin recognition complex, subunit 1-like	7.0E-06	1.0E-05	1.0E-05	8.0E-06
	TOP2A	Topoisomerase (DNA) II alpha	8.0E-07	1.0E-06	1.0E-06	6.0E-07
	RFC5	Replication factor C (activator 1) 5	2.0E-06	4.0E-06	4.0E-06	2.0E-06
	BLM	Bloom syndrome	7.0E-09	6.0E-09	4.0E-09	6.0E-09
**DNA repair**	DCLRE1C	DNA cross-link repair 1C (PSO2 homolog)	4.0E-07	6.0E-07	6.0E-07	4.0E-07
(8×10–18)	XRCC1	X-ray repair complementing defective repair	5.0E-07	8.0E-07	7.0E-07	4.0E-07
	MLH1	mutL homolog 1	2.0E-06	4.0E-06	4.0E-06	2.0E-06
	BRCA1	Breast cancer 1, early onset	2.0E-07	2.0E-07	3.0E-07	2.0E-07
	EXO1	Exonuclease 1	6.0E-08	7.0E-08	1.0E-07	6.0E-08
	TYMS	Thymidylate synthetase	1.0E-07	1.0E-07	2.0E-07	1.0E-07
	PMS2	Postmeiotic segregation increased 2	4.0E-05	8.0E-05	6.0E-05	5.0E-05
	FEN1	Flap structure-specific endonuclease 1	7.0E-06	1.0E-05	1.0E-05	8.0E-06
	NUDT1	Nudix-type motif 1	4.0E-05	7.0E-05	5.0E-05	5.0E-05
	RAD51	RecA homolog	1.0E-07	2.0E-07	2.0E-07	2.0E-07
**Apoptosis**	NUDT2	Nudix-type motif 2	2.0E-07	2.0E-07	1.0E-07	9.0E-08
(6×10–6)	CASP8AP2	CASP8 associated protein 2	2.0E-05	2.0E-05	2.0E-05	2.0E-05
	ITGB3BP	Integrin beta 3 binding protein	1.0E-05	2.0E-05	2.0E-05	2.0E-05
	TEGT	Testis enhanced gene transcript	4.0E-05	6.0E-05	5.0E-05	3.0E-05
	NDUFS1	NADH dehydrogenase (ubiquinone) Fe-S 1	3.0E-06	4.0E-06	4.0E-06	3.0E-06
	BNIP3L	BCL2/adenovirus E1B interacting protein 3-like	4.0E-05	6.0E-05	5.0E-05	4.0E-05
	SON	SON DNA binding protein	4.0E-05	7.0E-05	5.0E-05	4.0E-05
	CFL1	Cofilin 1 (non-muscle)	4.0E-05	6.0E-05	5.0E-05	3.0E-05
	GLO1	Glyoxalase I	4.0E-08	6.0E-08	7.0E-08	4.0E-08
	CHEK2	CHK2 checkpoint homolog	1.0E-08	8.0E-09	9.0E-09	1.0E-08
**RNA processing**	TTF2	Transcription termination factor 2	2.0E-08	2.0E-08	3.0E-08	3.0E-08
(4×10–8)	CSTF3	Cleavage stimulation factor, subunit 3	3.0E-07	5.0E-07	5.0E-07	3.0E-07
	HSPC148	Hypothetical protein HSPC148	1.0E-05	2.0E-05	2.0E-05	1.0E-05
	SNRPF	Small nuclear ribonucleoprotein peptide F	4.0E-05	6.0E-05	5.0E-05	4.0E-05
	DIS3	DIS3 mitotic control homolog	4.0E-06	8.0E-06	6.0E-06	5.0E-06
	SIP1	survival interacting protein 1	3.0E-05	4.0E-05	4.0E-05	3.0E-05
	FTSJ2	FtsJ homolog 2 (E. coli)	3.0E-06	4.0E-06	4.0E-06	3.0E-06
	CSTF2T	cleavage stimulation factor, subunit 2, tau	9.0E-07	2.0E-06	1.0E-06	7.0E-07
	LSM3	LSM3 homolog, U6 sn RNA associated	2.0E-06	3.0E-06	3.0E-06	2.0E-06
	SFRS1	splicing factor, arginine/serine-rich 1	2.0E-07	2.0E-07	3.0E-07	2.0E-07

Organized by GO categories, top hits by significance with p-values and binding ratios.

### Genes bound by E2F4 in both mouse and human capture cell cycle functional pathways

Because of the high conservation of the DREAM complex, we expected to observe in mouse a pattern of E2F4 binding similar to that found in human, with a common set of genes bound independent of the particular tissue, and enriched for proliferation-related pathways. To test this hypothesis, we performed E2f4 chromatin immunoprecipitations in seven primary mouse tissues and a mouse cell line using promoter microarrays representing 13,000 regions in the mouse genome (Figure 2B). As with human, E2f4 binds approximately 500 to 700 genes, the overlap between tissues is greater than 65% and as high as 85% (Figure 2B, Figure S6); using p-value cutoff of 10^−4^, a core set of approximately 450 genes are bound in common by E2f4 in all mouse tissues (Figure S7). The genes bound by E2f4 show functional pathway enrichment in cell cycle, proliferation, and DNA repair, as expected (Figure 2D).

To test whether the mouse genes bound by E2f4 were largely similar to those found in human, we identified the set of homologous genes present on both arrays and compared the core set of genes bound in each species (Figure 2C). To our surprise, we found that most of the genes bound by E2F4 were highly species-specific. Approximately 80% of the bound genes that had homologs in the second species were uniquely bound in the first species; only a fifth of the bound homologous genes shared E2F4 binding between species. Recent reports have indicated that transcription factor binding is preferentially conserved when a bound target gene is required for a transcriptional regulator's function [23]. Indeed, we determined that the genes bound in both species were enriched in known E2F4 functional categories like cell cycle control, proliferation, and DNA repair (Figure 2C). Interestingly, DNA packaging, which has been suggested to be a function of E2F4 in murine cells [27], was enriched as a functional category in the genes uniquely bound by E2f4 in mouse (Figure 2D, Figure S7).

### Gene expression programs in quiescent tissues can recover from removal of E2f4 during development

We tested whether the mouse genes bound by E2f4 in liver, kidney, and testes were dependent on the presence of E2f4 for proper transcription. We compared the gene expression patterns of these tissues in *E2f4* null mice to those found in identical tissues from E2f4 heterozygous littermates, which are phenotypically normal. During development, *E2f4* null mice have severe disruption of liver-based erythropoiesis [17]. In addition, *E2f4* null mice reproduce poorly, and this effect could be partially due to misregulation of gene expression in testicular tissues. Finally, kidney was chosen as a highly differentiated tissue that does not appear to have substantial proliferative capacity, and does not appear to be affected by removal of E2f4.

**Figure 3:**
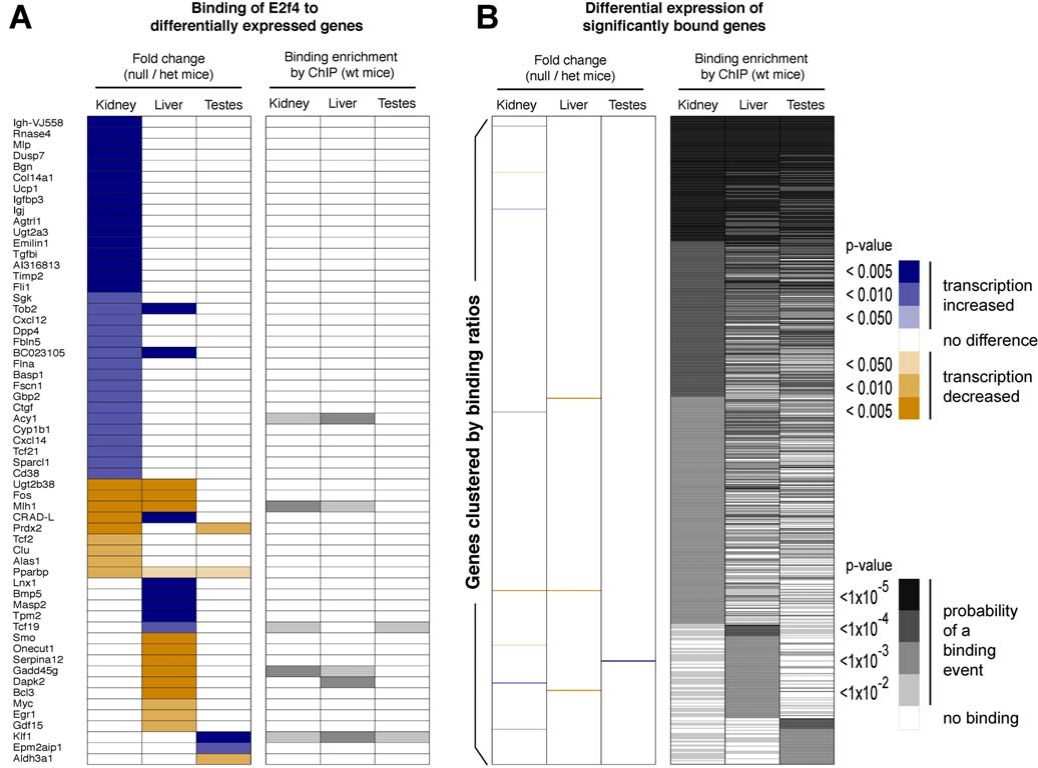
Gene expression changes upon germline removal of E2f4 transcription factor binding in mouse liver, kidney, and testes are minimal, and poorly overlap the genes bound in mice containing E2f4. (A) The complete list of genes that were differentially expressed when E2f4 was removed where the proximal promoter of these transcripts was present on the mouse promoter array. Genes were sorted by confidence in kidney, liver, and testes, sequentially, and the binding data then obtained separately. Most gene expression changes were tissue-specific, and very few of these genes showed in vivo E2f4 binding. (B) The complete list of genes bound by E2f4 in kidney, liver, and testes, clustered by transcription factor binding in each tissue sequentially. The transcripts of almost no genes bound in vivo were perturbed in the adult *E2f4* null mice.

All three mouse tissues had surprisingly minor transcriptional perturbations in the absence of E2f4. Specifically, *E2f4* null mice showed transcriptional perturbations among 10 genes in testes, 34 genes in liver, and 78 genes in kidney relative to heterozygous littermates (Figure 3, Figure S8). The genes whose transcripts showed changes in the absence of E2f4 were largely unique to each tissue (Figure 3A), and little overlap was observed between genes with altered expression and the genes bound by E2f4 in the same tissues (Figure 3B). We further considered the possibility that the E2f4-bound genes may show low-level gene expression changes that would be detectable if considered as a set [28]. We found no evidence of consistent up- or down-regulation in liver, kidney, or testes using any gene set combination reported to date, including the set of bound genes by E2f4 in mouse. In addition, the sets of differentially expressed genes were not enriched in any functional categories (not shown), and the promoters of these genes showed neither enrichment in direct E2f4 occupancy (Figure 3A) nor enrichment in the presence of E2f4 binding sequences when compared with the DNA present on the promoter arrays (Figure S4).

Altered transcripts in the *E2f4* null mouse tissues represent the final, stable result of compensation in mice that survive the removal of E2f4, and as such would be expected to include both direct and indirect E2f4 targets. Remarkably, the list of genes bound by E2f4 in adult wild-type tissues did not appreciably overlap with the genes whose transcription is perturbed by complete loss of E2f4 (Figure 3). As described above, E2f4 binding events rarely occur in regions not represented on the mouse promoter arrays; thus, the transcriptional perturbations in adult tissues affected by E2f4 removal during development are probably indirect. Given the high similarity to the phenotypically normal mice, it is reasonable to suggest that compensatory mechanisms involving other E2f family members largely rescue the *E2f4* null phenotype. The recovery of the tissue-specific gene expression programs underscores the well-known redundancy within the E2f-controlled cell cycle program [1], [4], [5].

## Discussion

We have combined gene expression analysis of mice lacking E2f4 with the conservation of transcription factor binding to dissect the conserved regulatory networks controlled by E2F4 that govern cell cycle and proliferation in primary mouse and human tissues.

### Tissue-independence of E2F4 binding and cell cycle control

Our genome-wide analysis reveals that within a single species, a core set of over 400 genes bound and potentially regulated by E2F4 are largely independent of the particular tissue inspected. High overlap between three in vitro human cell lines has been noted previously [8], [14], [15]; our report confirms and extends this result to three primary in vivo human tissues and an asynchronous human cell line. We performed similar experiments in an even more diverse set of primary tissues in mouse, ranging from brain to kidney to testes, and confirmed the tissue-independence of E2f4 binding in a second species. Because every primary tissue contains different sets of transcription factors, chromatin remodelers, and, indeed, chromatin structures, the presence of a commonly bound set of genes that makes up the large majority of E2F4 binding targets in multiple human (or mouse) tissues suggests that this key member of the DREAM complex binds independently of the above factors. Furthermore, the high overlap of E2F4 bound genes in the diverse human tissues we characterized indicates that the regulatory mechanisms used by the DREAM complex to control entry into cell cycle in human glioblastoma cells are likely employed in all human tissues [8]. Consistent with this, as expected the target genes shared commonly among multiple tissues in each species are functionally enriched in cell cycle, DNA repair, and DNA replication.

### Conservation of a binding event between mouse and human enriches functionally relevant genes

We took advantage of the genome-wide nature of our data in mouse and human to identify how conserved E2F4 binding is among the approximately five thousand homologous genes present on both microarrays. This approach revealed two key findings regarding how E2F4 controls cell cycle and proliferation via genomic binding. First, we found that less than a quarter of genes bound by E2F4 in one species were bound in the second. Our observation that E2F4 binding within a particular species is largely insensitive to tissue-specific binding partners and overall chromatin state makes the modest conservation of E2F4 binding events between species all the more remarkable. It has been suggested that the interaction of different members of the DREAM complex (e.g. E2f4 and p107) may vary between mouse and human; a variation that may have functional implications for E2f4's role in mouse [27]. Thus, one possible explanation is that variability with the composition and/or stoichiometry of the DREAM complex between mouse and human that is specific to each species may direct the complex to different sets of targets.

Second, a feature of the approximately fifty genes where E2F4 binding is conserved is their remarkable enrichment in cell cycle, proliferation, and DNA repair functions. This result is consistent with the hypothesis that when a transcription factor binding event is conserved, this conservation is a good indictor of a functional regulatory connection [23]. We predict that comparing genomic occupancy of a transcription factor in divergent species will be useful in general as a strategy to identify direct transcription factor targets.

### Cell cycle programs and tissue-specific transcription

We expected that removal of E2f4, as a key member of the DREAM complex would have profound implications for the correct gene expression of the genes bound by E2f4 in vivo. Instead, however, we found that developmental recovery and survival to adulthood corresponds with almost completely normal gene expression in multiple tissues, relative to their phenotypically normal heterozygous littermates. Given the substantial developmental defects described above, the subtle and limited changes in the gene expression profiles of *E2f4* null tissues is nevertheless surprising, despite the largely normal physiology of these mice in adulthood. It has been known since the report of viable *E2f4* null mice that the function of most tissues, even those directly impacted during development by absence of E2f4, recovers. Our findings reveal that this recovery extends to the level of gene expression; it appears that the absence of E2f4 can have profound, yet remarkably transient, implications for tissue-specific transcriptional programs. This recovery almost certainly depends on the overlapping roles that other members of the E2f family can play. For instance, it has been previously shown that E2F5 can largely compensate for the absence of E2F4 in vivo [22]. Fully understanding the complementary and often overlapping roles the E2f family play will require genomic and genetic dissection of genetically modified mice combinatorially lacking multiple E2f family members, using approaches such as those reported here.

Our results also provide direct support to the hypothesis that transcriptional binding is often neutral in nature [29]. Notably, the perturbed genes did not overlap significantly with E2F4 bound genes, and inspection of the perturbed sets of genes revealed no substantial functional category enrichment (including cell cycle and proliferation), thereby suggesting that these perturbations are not directly caused by removal of E2F4. Further supporting this observation, we found no enrichment of the canonical E2F4 binding sequence in the proximal promoter regions upstream of the genes perturbed by removal of E2F4. Taken together, it appears that E2F4 has a substantial number of binding events that are entirely dispensable for proper transcription of downstream genes, despite the well-characterized role it plays in cell cycle control.

### Conclusion

We have used comparative genomics approaches to explore the evolutionarily conserved regulatory pathways that E2F4, a key member of the DREAM complex, uses to maintain control of gene expression programs. We find that that most genes bound by E2F4 are common to numerous primary tissues within a species. This suggests that the regulatory architecture controlling quiescence may be similar among tissues that have remarkably different capacities for re-entry into the cell cycle. Understanding this architecture will require further studies to explore how E2f4 acts within specific cell types during development. The striking and specific conservation of E2F4 binding between mouse and human at cell cycle and proliferation genes suggests that only the targets crucial for the function of the DREAM complex are under selective pressure, yet our discovery that complete removal of E2f4 has at best modest effects on gene expression programs dramatically underscores the well-known redundancy within cell cycle regulation. Our study demonstrates the power of using the conservation of transcription factor binding at orthologous mouse-human genes as a tool to identify regulatory connections that appear to be under evolutionary pressure.

## Materials and Methods

### Reagents and antibodies

All chemicals were purchased from Sigma-Aldrich, and used as received unless otherwise noted. E2F4 antisera were obtained from Santa Cruz Biotechnology (polyclonal, rabbit, sc-1082) and used as described in prior studies [15], [27], or were created in the J. Lees laboratory (monoclonal mouse antisera LLF4-1) [30]. Data accession numbers at ArrayExpress are: E-TABM-272 and E-MEXP-1131.

### Mouse tissues for chromatin immunoprecipitation

Mice used in chromatin immunoprecipitation experiments were F1 males from a B6/C57 male cross with an A/J female (Jackson Laboratories). Islets suitable for ChIP studies were isolated by standard techniques and hand picking at Joslin Diabetes Center on mixed gender mice of the same genetic background (B6/C57xA/J). Other tissues were harvested using standard techniques, soaked or perfused with 1% formaldehyde, and homogenized in neutralization buffer followed by ChIP. For expression studies, E2f4 −/− homozygous and E2f4 +/− heterozygous knock-out mice were derived in a B6/C57 and 129S2/SvPas cross background [16], [31].

### Human tissues

Primary human hepatocytes were obtained from the Liver Tissue Procurement and Distribution Program (NIDDK contract number N01DK92310) at the University of Pittsburgh. Human pancreatic islets and pancreatic acinar tissues were the kind gifts of Gordon Weir, Abdulkadir Omer (Joslin Diabetes Center) and Nicolas Benshoff (University Minnesota) (NIDDK contract numbers NCRR ICR U4Z RR16606; U19DK6125).

### Mouse mRNA preparation for gene expression studies

Mouse tissues were harvested from two E2f4 −/− and two E2f4 +/− heterozygous littermates [16]. After organ removal, the tissues were homogenized, and the cells resuspended into Trizol with nuclease inhibitors. Corresponding mRNA from homozygotes and heterozygotes was obtained, normalized in concentration, and hybridized as biological duplicates to Affymetrix 430A Genome arrays using standard methods.

### Analysis of gene expression

The *limma* [26] and *affy* [32] packages within the R environment [33] were used to pre-process the array intensities and identify differentially expressed (DE) genes. The quality of the arrays was checked using exploratory data analysis methods [34]. Boxplots and density plots of unprocessed log-scale probe intensities were compared across arrays, and RNA digestion plots showing the 3′/5′ intensity ratios were used to check for similar rates of RNA degradation across arrays. Further quality diagnostics were based on the fit of a probe level model to the data, implemented using the *affyPLM* package [35], which models the dependence of probeset intensities on the probes and the array using robust regression procedures [36]. Chip pseudo-images of the signed residuals from the regression were plotted to check for spatial artifacts which may not appear in the raw-data image plots and plots of the Normalized Unscaled Standard Errors compared the fit of each array. Based on these quality checks, all arrays were retained for subsequent analysis. The probesets were then normalized and summarized using the Robust Multichip Average method RMA [37]. A linear model including empirical Bayes smoothing [26], [38] was fitted to the pre-processed data for all arrays to obtain moderated t-statistics (and B-statistics) corresponding to the contrast between heterozygous and E2f4 −/− for each tissue. The Benjamini-Hochberg correction BH [39] was applied to the p-values (corresponding to the moderated t-statistics) to give values adjusted for multiple testing across genes. By visual inspection of volcano plots, adjusted p-values of 0.05 were used to obtain sets of DE genes between E2f4 heterozygous and homozygous knock out mice. Permutation tests were used to check for systematic up-regulation or down-regulation of the genes bound by E2f4 in all tissues (as identified from the Microarray Analysis explained below). The permutation test is a simple non-parametric method for comparing the distributions of two sets, in this case the genes bound by E2F4 whose expression was analyzed, and the corresponding unbound genes.

### Chromatin immunoprecipitations

The procedure for chromatin immunoprecipitation has been reported previously [8], [14], [40]. Briefly, mice were sacrificed at 8–12 weeks and the following tissues were harvested: Brain, kidney, liver, pancreatic islets, pancreatic acinar, spleen, and testes. Both mouse and human tissues were treated with formaldehyde to covalently link transcription factors to DNA sites of interaction by either immersion in, or perfusion with 1% final concentration formaldehyde, followed by homogenization using a manual glass cell homogenizer. Chromatin in cell lysates was sheared by sonication at 4°C using a Misonix 3000 sonicator with power output set at 27–30 watts for ten 30-second pulses with one minute break intervals. The transcription factor-DNA complexes were enriched by chromatin immunoprecipitation, the cross-links reversed, and enriched DNA fragments and control genomic DNA fragments amplified using ligation-mediated PCR. The amplified DNA preparations, labeled with distinct fluorophores, were mixed and hybridized onto a promoter array with yeast tRNA and COT1 mouse or human DNA as non-specific carrier nucleic acids. A human genomic array (Hu19K) consisting of PCR products representing 19,000 proximal promoters was constructed to capture 1 KB of sequence immediately upstream of the transcription start sites (TSS) [15], [40]. In addition, the Hu19K array has additional coverage of 7 kb around the TSS of 200 transcription factors using 1 kb PCR fragments, as well as 4 kb coverage using 1 kb PCR fragments around all 250 known human microRNA loci. A similar mouse genomic array (Mm13K) was also used that represents 13,000 promoter regions, and coverage of mouse microRNA loci similar to the human array [41].

### ChIP-chip microarray analysis

The statistical analysis was performed using the *limma* package. Quality of the arrays was assessed using array images of the background and foreground intensities and also of the red/green (Cy5/Cy3) ratios to check for spatial artifacts. MA-plots (log-fold change against average log-intensity for each gene) [42] were used to compare different replicates for each tissue and the arrays that were most consistent with respect to quality and intensity levels were used for downstream analysis. The distribution of log-ratios across arrays varied in scale (after global median normalization and background correction). However, the scale variation was not observed to be associated with tissue type; rather the variation appeared to be more likely due to IP efficiency differences between individual experiments. The conclusions could only be reached because enough replicates were available for each tissue. Likewise, the replicates allowed sensible quality assessment and the best two arrays for each mouse tissue and three arrays for each human tissue were selected for subsequent analyses. Given the assumption that the log-ratio distributional differences were not associated with tissue type, the data were scale normalized between arrays using quantile normalization of the log-ratios (rather than a robust scale normalization, such as MAD scaling). Due to the one-probe-per-gene nature of the proximal promoter arrays, modified t-statistics were used to identify the probes that were enriched (bound) in each tissue. The corresponding BH adjusted p-values were used to correct for multiple testing across genes, and the results were visualized using volcano plots. Two thresholds were chosen by visual inspection of the volcano plots to define sets of binding sites of varying stringency for each tissue. To create lists of tissue-specific binding events, we used the stringent cut-off (adjusted p-value 10^−4^). The set of commonly bound genes was defined using the less stringent value of 10^−3^, to capture binding events falling just under the more stringent threshold in one or two tissues. To find orthologous mouse and human genes on the promoter arrays, gene symbols and accession numbers were linked to the Ensembl IDs for each species to match the two gene lists. The data in [15] were analyzed using the same pipeline described above for our data; binding sites were identified using an adjusted p-value of 10^−4^. The comparison with Litovchick ChIP data was done by comparing their published binding results to the cut-offs described in Figure S2 [8].

### Analysis of Gene Ontology GO categories

We determined the enrichment of functional categories among the bound gene sets using the GOstat tool [43], which detects significant enrichment of GO categories in a specific set of genes compared to the whole set of genes present on the array for each species. The method exploits Fisher's Exact Test to produce BH multiple-testing corrected p-values showing whether each GO term that appears in the selected group of genes is over-represented or under-represented.

### Analysis of the presence of E2F4 binding sequence

Given the sequences of the promoters on the ChIP array, a Perl script was used to automate BLAST searches of alignment across the genome and screen the BLAST output and BioPerl was used to extract the genomic coordinates of the promoters. To interrogate for the presence of the E2F4 motif, we used the one-kilobase E2F4 bound regions plus an additional 300 bases upstream and downstream to account for the resolution of the ChIP technique [41]. We used the E2F4 binding sequence as derived by [41], based on the positional weight matrix model [44] of binding specificity. The enrichment score of the presence of the binding motif was equivalent to the log-likelihood of these probabilities, and the threshold used was based on the possible maximum scores for each promoter. To confirm that the binding sequence results were not dependent on the particulars of the thresholds chosen, we chose a stringent threshold of 10.6 for the enrichment scores as well as a more lenient value of 10.0 (Supplemental Figure 4). To judge whether the E2F4 binding sites identified by our previous analysis had a significant over-representation of the E2F4 motif, the hypergeometric distribution was used to find the probabilities that those genes could have been randomly sampled from the genes on the promoter array. Based on the hypothesis of independence between the identified genes and the presence of the motif, the calculated p-values show the probability of the motif appearing in as many or more promoters as observed, compared to the background motif presence.

### Distance of E2F4 binding events to nearest transcriptional start site

Human E2F4 ChIPs in primary human liver were hybridized in duplicate to human whole chromosome 21 microarrays; mouse E2f4 ChIPs in primary mouse liver were hybridized in duplicate to mouse whole chromosome 16 microarrays (Agilent AMADID numbers 014841 and 015340, respectively). The *limma* package within R was used in the genomic microarray data analysis. Quality assessment included boxplots and images of the background and foreground intensities for both red (Cy5) and green (Cy3) channels, as well as MA-plots. All the arrays used in the analysis exhibited good quality. Expression red/green log-ratios were median-normalized, after background subtraction, within each array. E2F4 binding events were empirically assigned as minimum contiguous regions comprising, at least, one probe for which the red/green ratio was greater than 5, or two adjacent probes with ratios greater than 2.5 and a combined (summed) ratio greater than 6.5, or three adjacent probes with ratios greater than 2 and a combined (summed) ratio greater than 9. A Perl script was used to compute the genomic base pair distance between each binding event and the closest transcription start site. Transcriptomic annotation relied on tables downloaded from the UCSC Genome Browser server. The smoothed histograms in Figure S1 were generated in R by applying the *density* function (default parameters) to the distance distributions and plotting the respective outcomes.

## Supporting Information

Figure S1 (0.05 MB PDF)

Figure S2 (0.06 MB PDF)

Figure S3 (0.09 MB XLS)

Figure S4 (0.17 MB PDF)

Figure S5 (0.13 MB PDF)

Figure S6 (0.08 MB PDF)

Figure S7 (0.14 MB XLS)

Figure S8 (0.08 MB XLS)
