# Supplementary material for: Cell Cycle Genes Are the Evolutionarily Conserved Targets of the E2F4 Transcription Factor
Source: PLoS One. 2007 Oct 24;2(10):e1061. doi: 10.1371/journal.pone.0001061 (PMC2020443; doi:10.1371/journal.pone.0001061)
Supplement: Figure S1 — (0.05 MB PDF) [file pone.0001061.s001.pdf]

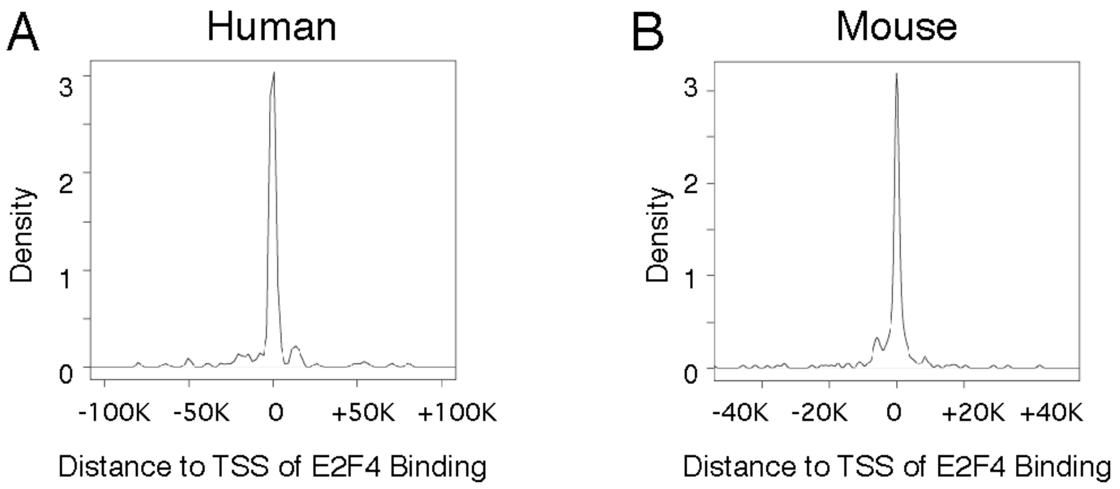

Supplemental Figure 1. Distance between E2F4 binding sites and nearest transcription start sites. E2F4 ChIPs were hybridized to whole chromosome arrays, and the average distance between each E2F4 binding event and the nearest annotated transcriptional start site was calculated (see Methods). The results of plotting the distances as a density plot are shown for (A) a E2F4 ChIP in human liver on human chromosome 21, and (B) a mouse liver ChIP on mouse chromosome 16. Both data sets indicated that almost all E2F4 binding events occur within 1 kb of a transcription start site.
