# Supplementary material for: Cell Cycle Genes Are the Evolutionarily Conserved Targets of the E2F4 Transcription Factor
Source: PLoS One. 2007 Oct 24;2(10):e1061. doi: 10.1371/journal.pone.0001061 (PMC2020443; doi:10.1371/journal.pone.0001061)
Supplement: Figure S2 — (0.06 MB PDF) [file pone.0001061.s002.pdf]

A p-value = 1E-03

	Liver	HepG2	Islets	Acinar
Acinar	0.73	0.72	0.79	805
Islets	0.84	0.78	725	
HepG2	0.75	800		
Liver	849			

Fraction Overlap
< .60
0.60 - 0.69
0.70 - 0.79
> 0.80

B p-value = 1E-04

	Liver	HepG2	Islets	Acinar
Acinar	0.73	0.72	0.80	502
Islets	0.84	0.77	454	
HepG2	0.73	516		
Liver	520			

C p-value = 1E-05

	Liver	HepG2	Islets	Acinar
Acinar	0.75	0.74	0.77	273
Islets	0.89	0.72	265	
HepG2	0.72	288		
Liver	302			

Supplemental Figure 2. The overlap among the genes bound by E2F4 in multiple human tissues does not depend on cutoff chosen. Each panel represents the overlap in binding data between the tissues located in the row-column combination, as labeled at p-values of (A) 1 x 10^{-3}, (B) 1 x 10^{-4}, and (C) 1 x 10^{-5}. The total number of genes bound in each tissue, given the p-value cutoff, is shown as the white background diagonal that represents the same tissue row-coumn (e.g. acinar versus acinar, et cetera).
