# Supplementary material for: Cell Cycle Genes Are the Evolutionarily Conserved Targets of the E2F4 Transcription Factor
Source: PLoS One. 2007 Oct 24;2(10):e1061. doi: 10.1371/journal.pone.0001061 (PMC2020443; doi:10.1371/journal.pone.0001061)
Supplement: Figure S4 — (0.17 MB PDF) [file pone.0001061.s004.pdf]

P-Value of GO Category Enrichment in Tissue-Specific E2F4 Binding Events

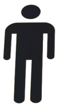

	Cell Cycle<br>GO:0007049	DNA Replication<br>GO:0006260	DNA Repair<br>GO:0006281	DNA Packaging<br>GO:0006323	RNA Processing<br>GO:0006396	Apoptosis<br>GO:0006915	Cell Differentiation<br>GO:0048468
**Liver (62)**	> 0.01	> 0.01	> 0.01	> 0.01	> 0.01	> 0.01	> 0.01
**Islets (20)**	> 0.01	> 0.01	> 0.01	> 0.01	0.076	> 0.01	> 0.01
**Acinar (90)**	0.005	> 0.01	3.03E-05	> 0.01	> 0.01	> 0.01	> 0.01
**HepG2 (100)**	> 0.01	> 0.01	> 0.01	> 0.01	0.001	> 0.01	0.0071

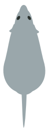

	Cell Cycle<br>GO:0007049	DNA Replication<br>GO:0006260	DNA Repair<br>GO:0006281	DNA Packaging<br>GO:0006323	RNA Processing<br>GO:0006396	Apoptosis<br>GO:0006915	Cell Differentiation<br>GO:0048468
**Liver (36)**	> 0.1	> 0.1	> 0.1	> 0.1	> 0.1	> 0.1	> 0.1
**Kidney (19)**	0.035	> 0.1	> 0.1	> 0.1	> 0.1	> 0.1	> 0.1
**Testes (33)**	0.052	> 0.1	> 0.1	> 0.1	> 0.1	> 0.1	> 0.1
**Spleen (31)**	0.071	> 0.1	> 0.1	> 0.1	> 0.1	> 0.1	> 0.1
**Acinar (85)**	> 0.1	> 0.1	> 0.1	> 0.1	> 0.1	> 0.1	> 0.1
**Islets (63)**	> 0.1	> 0.1	> 0.1	> 0.1	> 0.1	> 0.1	> 0.1
**Brain (16)**	> 0.1	0.028	> 0.1	> 0.1	> 0.1	> 0.1	> 0.1
**Min6**							

Supplemental Figure 4. Tissue-specific E2F4 binding events are not significantly enriched in functional pathways in either mouse or human. The known functional pathways regulated by E2F4 are shown here; other pathways are similarly unenriched (not shown).
