# Supplementary material for: Cell Cycle Genes Are the Evolutionarily Conserved Targets of the E2F4 Transcription Factor
Source: PLoS One. 2007 Oct 24;2(10):e1061. doi: 10.1371/journal.pone.0001061 (PMC2020443; doi:10.1371/journal.pone.0001061)
Supplement: Figure S5 — (0.13 MB PDF) [file pone.0001061.s005.pdf]

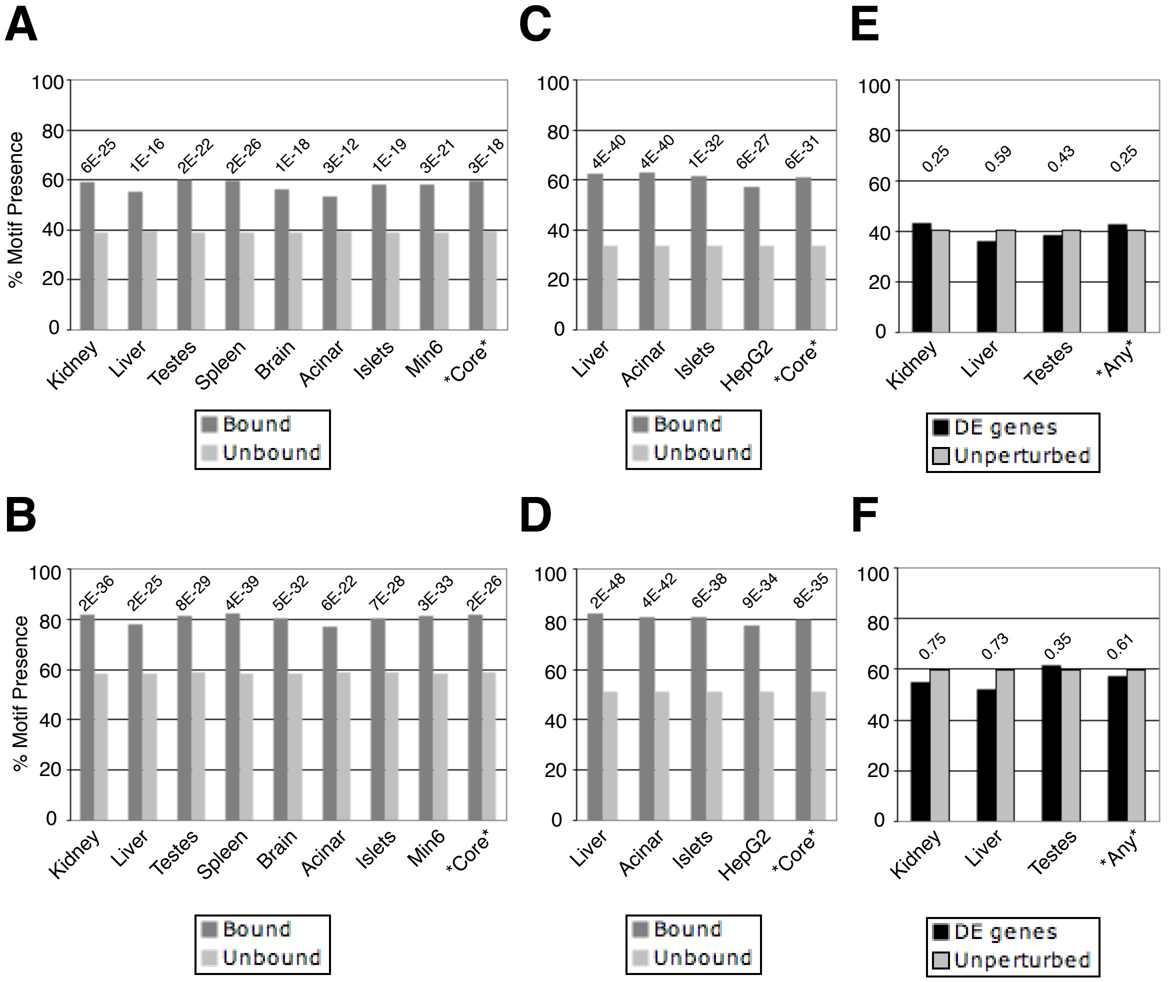

Supplemental Figure 5. Presence of the E2F4 binding motif in promoter regions. Percent of bound and unbound promoters containing the canonical E2F4 motif is shown for mouse and human tissues at two cut-off values. Notably, the E2F4 motif is a short sequence that occurs are high frequency in the genome, as evidenced by the high occurrence of the sequence in unbound regions, regardless of the binding cutoff. The promoter arrays used here represented approximately one kilobase of sequence; because of the spatial resolution of the ChIP assay, for interrogation of binding site presence an additional 300 bases were computationally added to both ends of the represented region. Significance of motif enrichment in the bound or differentially expressed promoters is given as a p-value above the columns for each tissue. (A) Mouse stringent cut-off = 10.6. (B) Mouse lenient cut-off = 10.0. (C) Human stringent cut-off = 10.6. (D) Human lenient cut-off = 10.0. (E) Differentially expressed vs. unperturbed, cut-off = 10.6. (F) DE vs. unperturbed, 10.0.
