# Supplementary material for: Cell Cycle Genes Are the Evolutionarily Conserved Targets of the E2F4 Transcription Factor
Source: PLoS One. 2007 Oct 24;2(10):e1061. doi: 10.1371/journal.pone.0001061 (PMC2020443; doi:10.1371/journal.pone.0001061)
Supplement: Figure S6 — (0.08 MB PDF) [file pone.0001061.s006.pdf]

A **p-value = 1E-03**

	Kidney	Liver	Testes	Spleen	Brain	Acinar	Islets	Min6
Min6	0.71	0.70	0.77	0.71	0.73	0.65	0.70	1165
Islets	0.65	0.65	0.69	0.67	0.67	0.62	1100	
Acinar	0.70	0.73	0.69	0.71	0.71	1079		
Brain	0.86	0.81	0.83	0.83	1131			
Spleen	0.85	0.77	0.83	1152				
Testes	0.84	0.80	911					
Liver	0.82	1140						
Kidney	1083							

Fraction Overlap
< .60
0.60 - 0.69
0.70 - 0.79
> 0.80

B **p-value = 1E-04**

	Kidney	Liver	Testes	Spleen	Brain	Acinar	Islets	Min6
Min6	0.73	0.72	0.77	0.76	0.75	0.63	0.74	634
Islets	0.73	0.73	0.69	0.75	0.75	0.69	570	
Acinar	0.70	0.74	0.71	0.72	0.73	634		
Brain	0.86	0.82	0.85	0.86	657			
Spleen	0.84	0.80	0.86	684				
Testes	0.85	0.79	549					
Liver	0.80	657						
Kidney	655							

C **p-value = 1E-05**

	Kidney	Liver	Testes	Spleen	Brain	Acinar	Islets	Min6
Min6	0.75	0.75	0.79	0.76	0.82	0.70	0.77	340
Islets	0.76	0.75	0.74	0.80	0.80	0.79	310	
Acinar	0.73	0.72	0.73	0.72	0.73	374		
Brain	0.88	0.84	0.85	0.85	384			
Spleen	0.83	0.77	0.82	362				
Testes	0.83	0.80	332					
Liver	0.80	367						
Kidney	353							

Supplemental Figure 6. The overlap among the genes bound by E2F4 in multiple mouse tissues does not depend on cutoff chosen. Each panel represents the overlap in binding data between the tissues located in the row-column combination, as labeled at p-values of (A) 1×10^{-3}, (B) 1×10^{-4}, and (C) 1×10^{-5}. The total number of genes bound in each tissue, given the p-value cutoff, is shown as the white background diagonal that represents the same tissue row-column (e.g. acinar versus acinar, et cetera).
